# Studies on the Use of Loan Extraction to Produce Natural Shower Gels (Cosmetic) Based on Grape Pomace Extracts—The Effect of the Type of Surfactant Borrowed

**DOI:** 10.3390/molecules30183709

**Published:** 2025-09-12

**Authors:** Tomasz Wasilewski, Zofia Hordyjewicz-Baran, Katarzyna Malorna, Ewa Dresler, Ewa Sabura, Maciej Zegarski, Natalia Stanek-Wandzel

**Affiliations:** 1 Łukasiewicz Research Network-Institute of Heavy Organic Synthesis “Blachownia”, Energetykow 9, 47-225 Kedzierzyn-Kozle, Polandnatalia.stanek@icso.lukasiewicz.gov.pl (N.S.-W.); 2 Department of Cosmetology, Faculty of Medical and Health Sciences, University of Radom, Chrobrego 27, 26-600 Radom, Poland; 3 Onlybio.Life S.A., Jakóba Hechlińskiego 6, 85-825 Bydgoszcz, Poland; maciej.zegarski@onlybio.life

**Keywords:** grape pomace, cosmetics, surfactants, micellar extraction, loan extraction

## Abstract

The growing interest of consumers in natural products contributes to the increasingly widespread use of plant extracts as carriers of active ingredients in cosmetic formulations. Among plant materials, grape pomace, which remains after wine production, is of particular importance due to its known high bioactive compounds content. Micelle-assisted extraction was used to effectively extract these compounds. The aim of this study was to investigate the potential of various surfactants in the extraction process for cosmetic application. It was particularly important that the surfactants were borrowed from the final formulation of the designed cosmetic preparations. The concept of loan extraction for the production of cosmetics was described. The influence of the type of surfactants on the extraction efficiency was assessed by determination of individual phenolic compounds, amino acids and sugars using LC-MS/MS, as well as by determination of the total phenolic content and antioxidant activity using UV-VIS. The results obtained confirmed that the type of surfactants has a significant impact on extraction efficiency. The studies conducted proved that the application of the concept of loan extraction in the production of hygiene cosmetics, as exemplified by shower gels, enables the production of safe and natural products with reduced skin irritant potential.

## 1. Introduction

Cosmetic products are complex mixtures of ingredients with varying physical, chemical, and functional properties, developed to achieve the desired performance and quality [1,2,3,4,5,6]. Today, the cosmetics industry is a diverse and dynamic market with growing public interest, particularly in plant-based ingredients. Additionally, the cosmetics market is expanding due to innovations from the scientific community, which encourages further progress and growth in this field. In a competitive global market, the most cost-effective methods for developing cosmetic formulations are preferred.

Current consumer demands and international regulations are forcing the cosmetic industry to search for new active ingredients from renewable natural sources to produce greener and safer products. Botanical extracts provide an almost unlimited source of these new active ingredients [7]. This, in turn, leads to a research and development strategy in the cosmetics industry focused on sourcing “green” ingredients for the production of modern products and the reformulation of existing ones. Such efforts are necessary to meet contemporary consumer expectations for environmental sustainability while maintaining the efficacy of final products, which is an extremely challenging task [8,9].

Moreover, the shift towards more “natural cosmetics” has also become a requirement under current global regulations, which have banned many traditional chemicals from being used in the manufacture of products for human use. These regulations recommend the gradual replacement of this chemicals with other compounds, preferably derived from renewable natural sources [10,11]. Plants provide a wide range of active ingredients that are desirable in cosmetics and can be used in a variety of applications, such as protecting against UV radiation and pollution, formulating creams, creating fragrances, and mitigating the effects of skin aging [7].

Increased interest in natural products has led to the growing use of plant extracts as active ingredients in cosmetics. A cosmetic can be considered “green” if its formula contains active ingredients of plant origin, such as minerals and natural chemical compounds, rather than synthetic counterparts. Ideally, it should be produced in an environmentally sustainable manner, using processing methods that respect nature and adhere to organic farming principles [8,9,10,11,12,13,14].

Plant materials represent one of the oldest source of bioactive compounds, which are known for their numerous beneficial biological effects, including antioxidant, antimicrobial, anti-enzymatic and anti-inflammatory properties. Moreover, plant constituents are usually complex mixtures of bioactive substances that are challenging to replace with synthetic analogs. Bioactive plant compounds include a wide range of components, including phenolic compounds (such as phenolic acids and flavonoids), terpenes, steroids, carotenoids, sterols, saponins, fatty acids, carbohydrates, and peptides [15].

Increased consumer demand for conscious, sustainable and beneficial products has prompted researchers from both industry and universities around the world to search for new strategies capable of reducing the environmental footprint, particularly regarding industrial waste. Among the various by-products commonly considered waste, those obtained from the wine industry have attracted the attention of a wide variety of companies beyond wineries. In particular, grape pomace is noteworthy because of its high content of bioactive molecules, especially phenolic compounds [16,17,18,19,20,21,22]. These compounds can be recovered from grape pomace and used as active ingredients in cosmetic products. The use of bioactive ingredients found in grape pomace to produce high-value cosmetics can contribute to the development of innovative products and the expansion of the grape value chain [23,24,25,26,27,28]. The variety of grape types, geographic locations, and even the distribution of the same varieties, along with winemaking methods, affect the abundance and diversity of the composition of these raw materials [29,30].

Studies have shown that red grape pomace (RGP) and white grape pomace (WGP) differ in both the profile of phenolic compounds and total phenolic content, depending on the grape variety and terroir conditions. RGP is characterized by an abundance of stilbenes, such as resveratrol, as well as phenolic acids, flavanols and anthocyanins. WGP, on the other hand, is distinguished by its high content of various phenolic acids, flavanols (such as catechin and epicatechin), as well as flavonols (including quercetin) and flavonoid aglycones. Both types of pomace contain similar amounts of some compounds, such as caffeic acid, catechin, and its isomer, epicatechin, but overall RGP shows a higher content of polyphenols compared to WGP [31,32,33,34].

Various extraction methods can be used to fractionate and isolate valuable substances from plant material. Known phenolics extraction techniques include: solid-phase extraction (SE) [35,36,37], ultrasonic, microwave-assisted extraction (MAE) [38,39,40,41,42], pressurized liquid extraction (PLE) [43,44,45,46,47], enzyme-assisted extraction (EAE) [48,49,50,51,52,53] and supercritical fluid extraction (SFE) [54,55]. SPE methods are particularly popular because they are simple to use and require short extraction times.

Although there are various extraction technologies, steam/water distillation and organic solvent extraction—along with their with appropriate adaptations—remain the primary industrial methods for extracting non-volatile and volatile fractions [56,57,58,59,60,61]. Many extraction process use surfactants as effective tool for effective extraction [62,63,64].

This article presents an innovative approach to cosmetic production, emphasizing the key role of “loan extraction”.

The idea behind Loan Extraction (LE) is to develop and use an extraction medium with specific properties, consisting exclusively of components borrowed from the composition of the product to be manufactured. The resulting extract is then added in its entirety to the cosmetic product. As a result, the composition of the manufactured product contains no additional components other than those isolated from the plant material [65,66]. Using the idea of LE, various conventional extraction methods can be applied to make cosmetics, including micellar extraction, solvent extraction or ultrasound-assisted extraction.

In accordance with the concept of LE, the extraction medium in the present study is an aqueous solution that contains compounds which are components of the shower gel being produced. The borrowed ingredients: surfactants and preservatives are used for the extraction process and, once the process is complete, they return to the final cosmetic formulation enriched with bioactive compounds leached from the plant material. Contrary to conventional practices, in which extracts are typically introduced during the main stages of mass cosmetics production, LE redefines the manufacturing process by incorporating the extraction stage as an integral part of cosmetics production.

Surfactants, with their amphiphilic structure, are a promising alternative for improving the extraction of bioactive compounds from plant material. These amphiphilic compounds have a unique ability to reduce surface and interfacial tension, enabling them to interact effectively with both polar and non-polar substances. The physicochemical properties of surfactants enable them to participate in the extraction of a wide range of bioactive compounds from complex biological sources. In addition, the ability of surfactants to form micelles in aqueous solutions can significantly increase the solubility of hydrophobic substances. Although the use of surfactants offers multiple benefits for selective solubilization and increased extraction efficiency, the use of surfactants also requires careful consideration of parameters that have a significant impact on environmental aspects [67,68]. In view of the growing global demand for natural products, there is an increasing focus on the development of extraction methods that are both selective and address aspects such as sustainability, ease of use and safety for humans and the environment. In the context of wide cosmetic applications, it is important to select surfactants taking into account their selectivity as well as biodegradability, naturalness index, irritation potential and renewable origin.

In our previous study, we investigated extracts obtained through LE from grape pomace, a by-product of wine production. The composition of the extraction medium was carefully selected based on the designed final cosmetic product to produce aggregates (micelles) in the bulk phase. This targeted composition facilitated efficient leaching of cosmetically valuable bioactive ingredients from the plant material [65,66].

The present study compared the effectiveness of extraction media composed of borrowed surfactant compounds, exclusively prepared from components contained in the formulation of the cosmetic under development. The extraction media involved aqueous solutions of various types of surfactants with the aggregates (micelles) formed in the bulk phase. The emphasis was on demonstrating the benefits of incorporating this type of extraction into the production of hygienic cosmetics. White grape pomace was used as the plant material for this innovative extraction method.

The development of surfactant-based delivery systems is targeting a wide range of applications, including the delivery of active compounds in cosmetics.

Surfactant-based carriers play a key role in the solubilization and delivery of functional ingredients. Their incorporation into cosmetic products improves sensory properties, enables controlled bioactive release, enhances product texture, promotes skin penetration, and provides targeted action in specific areas, thereby maximizing health benefits.

This article is focused on application of surfactants for efficient leaching of bioactive molecules from plant material during the extraction process.

## 2. Result and Discussion

### 2.1. Development of Loan Extraction Process to Obtain Cosmetically Valuable Compounds from Waste Plant Material, Using Borrowed Components from the Final Product

#### 2.1.1. Extraction Process

The initial objective of the research was to develop human- and environmentally safe cosmetics enriched with bioactive compounds derived from plant extracts. The proposed cosmetic preparation method includes a key step described as “Loan Extraction” (LE), which is defined as “extraction using ingredients borrowed from the final product.” With such pragmatics, there is no longer a need for additional purification of the extract obtained, which contributes to reducing the introduction of additional chemicals into the environment thereby increasing human and environmental safety.

For this purpose, it was planned to develop an effective micellar mediated extraction medium that contains only ingredients intended for use in the final product. These ingredients were given a dual function: both as a cleansing agent in final hygiene cosmetics and as an effective extraction medium. It is important to note that valuable cosmetic substances contained in plant materials can be either hydrophilic or hydrophobic. Hydrophobic compounds remain insoluble in aqueous solutions, thus their extraction can be problematic. To increase the efficiency of their effective extraction, a surfactant compound from the final cosmetic composition was chosen for the extraction process. Surfactants, due to their amphiphilic properties, form associative aggregates (micelles) in aqueous solutions. Inside these aggregates, hydrophobic substances, such as bioactive compounds extracted from the plant material, can be solubilized [65].

In the first step a formulation of the model hygienic cosmetics (shower gels) were designed.

Commercially available shower gels are water-based formulations in which anionic surfactants serve as the primary functional agents. Amphoteric and nonionic surfactants are also usually added to improve the performance properties. Typically, the formulation contains about 10–20% surfactants. Due to structure and adsorption properties, surfactants are responsible for the washing effect generated by shower gels. To ensure that the finished product meets consumer expectations in terms of functionality, cosmetic formulations typically include pH regulators, viscosity modifiers, fragrance compositions, and colorants [69]. Additionally, cosmetics may be enriched with ingredients that enhance their quality and distinguish them from other products on the market. Currently, several key trends are dominated, including the increasing popularity of multifunctional ingredients that work on multiple levels, such as plant extracts [1].

To design model shower gel for research, commercial surfactants were used. These surfactants were selected in accordance with current trends regarding consumer desire for natural products. All ingredients used were EcoCert and COSMOS compliant. This is important because consumers today are demanding cosmetic products with biodegradable ingredients and packaging, and brands are using claims that their products contain “naturally derived,” “natural” or “organic” ingredients in response to consumer demands. These claims can be supported by various certification standards (e.g., Ecocert, COSMOS and NATRUE standards) [70].

The target formulation of designed showe gel is shown in Table 1.

Surfactants are among the most used ingredients in hygiene products. The appropriate selection of surfactants in terms of type and ratio is crucial in determining the cleansing efficacy and foam quality of a product. Sodium Coco-Sulfate, Disodium Cocoyl Glutamate, Decyl Glucoside and Cocamidopropyl Betaine were selected as surfactants in the formulation of the shower gel to ensure its gentle and safe properties. What is very important the chosen surfactants are derived from natural sources. Sodium Coco-Sulfate and Cocamidopropyl Betaine are derived from coconut oil, Disodium Cocoyl Glutamate from fermented vegetables. This makes them a more sustainable, less skin irritation and renewable option compared to some synthetic surfactants [71,72].

**Figure 1:**
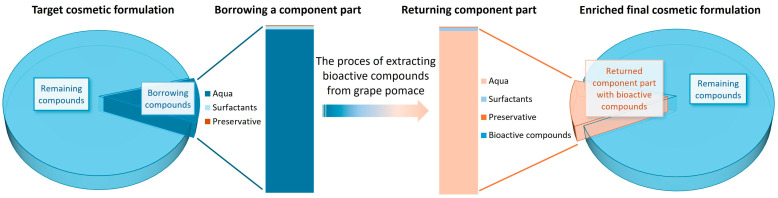
Schematic diagram of the Loan Extraction concept.

Surfactants intended for use in cosmetic preparations to facilitate the washing process were ‘borrowed’ in form of their preserved aqueous solution and used for extraction purposes. The process of micelle-assisted extraction is based on the same principle of action of surfactants in extracting bioactive compounds as in dirt removal. The aim of the research was to empirically determine the type of surfactant that would enable the most effective extraction of bioactive compounds from plant material. The range of ingredients used for extraction constituted 10% of the composition of the shower gel, which will ultimately return to the formulation in the form of an extract. The final product will be enriched with bioactive compounds from plant extract. The idea of Loan Extraction process is presented in Figure 1.

**Figure 2:**
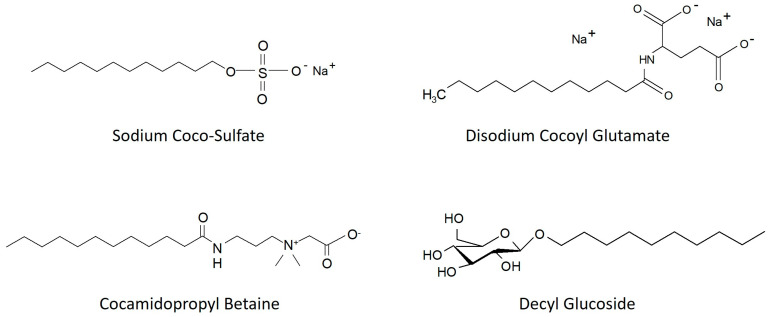
Chemical structures of the surfactant components.

The borrowed components were selected in terms of functionality and safety so they can be used directly in end user products such as cosmetic formulations. Different group and structure of surfactants were chosen for the investigation. The borrowed components and used in the extraction process are presented in Table 2. The chemical structures of the surfactant components are presented in Figure 2.

The extraction conditions were chosen based on our previous experience. The extraction conditions used are as follows: ratio of grape pomace to extraction medium: 4:1, time: 20 min, temperature: 22 °C.

The chemical structure of surfactants contributes to their surface active properties. This phenomenon results from the reduction in the surface tension in aqueous solutions and the interfacial tension in systems involving immiscible liquids. An essential characteristic of surfactants is their capacity to self-assemble in solution, forming the organized structures known as micelles. Micelles are generated one the surfactant concentration exceeds a specific concentration limit, called the critical micellization concentration, CMC. Various compounds can be solubilized inside micelles, which forms the background of the micelle extraction method [73]. Using aqueous surfactant solution, instead of harmful organic solvents it is possible to solubilize the bioactive compounds from plant material. Apart from avoiding the use of toxic organic eluents like methanol, propanol and ethyl acetate, the primary advantages of this methodology include a low extraction time and cost-effective processing [65]. In addition, the use of different micellar systems makes it possible to increase the efficiency of the extraction process of compounds that are poorly or not soluble in pure water. Furthermore, the surfactants used in the extraction process are derived from the composition of the cosmetic product, as they act as integral solubilizing agents. As a result, aqueous solutions of surfactants can improve the extraction process by eliminating the need for additional purification steps, solvent evaporation or significant energy consumption. This approach can also increase the recovery efficiency of bioactive phytochemicals, such as polyphenols, surpassing the results achieved with conventional solvents [74].

The process is completely safe, non-toxic to humans and environmentally friendly, and thus belongs to the group of methods that meet the principles of “green chemistry”. Micellar extraction is ideal for the cosmetics industry, as the surfactants are commonly used in final products and after the extraction processes they can be used directly in the cosmetic formulation, enriched with extracted compounds so in this way the process can be considered waste-free.

The properties of polyphenolic compounds affect the antioxidant properties of plant extracts determining their potential anti-inflammatory and regenerative effects on the skin. For this reason, these raw materials are successfully used in cosmetic preparations. In our study, we used a by-product material—grape pomace—resulting from wine production. Grapes are known to be rich in polyphenolic compounds, and pomace is a reservoir of these compounds, not used in the winemaking process. Winemaking residues represent a low-cost and vibrant source of bioactive compounds since more than 70% of grape phenolics remain in the pomace [75,76]. The increasing scientific and industrial focus on valorizing winemaking waste and efficiently recovering polyphenols is driven by the substantial generation of grape pomace, the potential economic benefits, and the resulting reduction in environmental impact [22,77].

Sodium Coco-Sulfate represents anionic surfactants derived from coconuts with a negatively charged head group. It is a biobased homolog of the commonly employed surfactant sodium lauryl (dodecyl) sulfate. Anionic surfactants are widely used in cleansing products, such as shampoos, body washes and facial cleansers. They are effective at removing dirt, oil and other impurities from the skin.

Disodium Cocoyl Glutamate is a biodegradable anionic surfactant produced from L-glutamic acid (an amino acid) and vegetable fatty acids from coconut oil. It is considered to be the safest and mildest as indicated by the MTT_50_ test and red blood cell (RBC) test, respectively, from chosen surfactants [78].

Cocamidopropyl Betaine is a quaternary ammonium salt of natural origin that is widely used in cosmetic and skincare products for several purpose, such as surfactants and viscosity control [72]. It is an amphoteric surfactant, which means it has both a positively charged (cationic) and a negatively charged (anionic) part of its structure. This amphoteric nature allows it to function as a surfactant and contribute to the foaming and cleaning properties of various personal care and cleaning products. Cocamidopropyl betaine is generally considered biodegradable, meaning that it can break down in the environment over time.

Decyl Glucoside represents non-ionic surfactants from alkyl polyglucosides—the most important group of sugar based surfactants. They have a glucose head group derived from corn, and a fatty alcohol tail group that is derived mainly from palm kernel oil. Apart from their renewable feedstock, they are often preferred as surfactants due to their superior biodegradability, dermatological and ocular safety [79]. In the personal care industry, they act as cleansing agents, foam stabilizers, and rheology modifiers [80].

The amount of natural ingredients in a product is indicated by the Natural Origin Index (NOI). The ISO 16128 standard [81,82] defines the NOI and outlines the methodology for calculating it for cosmetic ingredients and finished products. This standard aims to harmonize global markets for natural and organic cosmetics. An ingredient is assigned a NOI of 1 if it is classified as a natural ingredient, while those that do not meet this classification receive a NOI of 0. Natural ingredients include plant-based components and water. Derived natural ingredients, which are modifications of natural ingredients, have an NOI that ranges between 0% and 100%.

To calculate the total NOI of a product, one must multiply the percentage contribution of each component of the product by its corresponding value of NOI. Subsequently, by summing all the obtained values, the total NOI of the product can be determined. The NOI values utilized for this calculation are sourced from the manufacturers of the respective raw materials.

In order to assess the irritating potential of surfactants and finished products containing these compounds on human skin, in vitro and in vivo tests are used [83,84,85,86]. In this study, the irritating potential was determined using an in vitro method, namely the zein test. The name of the test comes from the protein used—zein, which mimics the structure of human skin proteins. This method is based on the analysis of the solubilization of zein, which is practically insoluble in water but solubilizes in the presence of surfactants. This solubilization is expressed by determining the nitrogen content (based on the Kjeldahl method) in the dissolved proteins, which refers to the so-called Zein Number and is the amount of nitrogen expressed in milligrams contained in the dissolved protein after contact with 100 mL of the test solution in which protein solubilization occurred.

This method is particularly important because nitrogen is a key component of proteins, which are the basic structural elements of the skin. The result of the analysis, i.e., the Zein Number, is used to classify substances in terms of their irritating potential: substances with a zein number below 200 mgN/100 mL are considered non-irritating, from 200 to 400 mgN/100 mL as moderately irritating, and above 400 mgN/100 mL as highly irritating [87].

Table 3 presents the parameters considered for the surfactants investigated in this study.

Decyl Glucoside, Sodium Coco-Sulfate and Disodium Cocoyl Glutamates are the most sustainable, with 100% NOI values.

The solubility of a poorly soluble solute depends on the surfactant concentration and is low when its concentration is below the critical micellar concentration. Above the CMC, the solubility of the solute increases linearly with increasing surfactant concentration [92,93,94].

In the present study, a surfactant concentration of 2% (*w*/*w*) was employed for the preparation of micellar extraction medium, which significantly exceeds the critical micelle concentration values of the surfactants utilized. The CMC values of the individual surfactants are presented in Table 3. A lower CMC value indicates an enhanced capacity for surfactant self-association, thereby facilitating the formation of micelles capable of effectively solubilizing hydrophobic compounds within their structure. The lowest CMC values was representing by CB, the highest for SCS. However, selection of the concentration for extraction process much above the CMC ensures the presence of micelles in the extraction medium for each surfactant system applied. A meticulous analysis of surfactant concentration and micelle formation processes is critical for the efficacy of the extraction process, thereby validating the suitability of the chosen surfactant system for applications in micellar extraction media. A significant exceedance of the CMC value by the applied surfactant concentration ensures that the required threshold for effective micelle formation has been achieved, which translates into the effectiveness of the selected extraction media for solubilizing lipophilic compounds derived from plant material.

The CMC values are related to the parameter known as the Zein Number. Zein is a hydrophobic protein that exhibits no solubility in water in the absence of surfactants. Studies on the interactions between surfactant systems and zein protein, which lead to its denaturation, contribute to the assessment of the irritant potential of the compounds used.

A higher value of Zein Number indicates a greater interaction of surfactants with proteins, which is interpreted as an increase in irritating potential. The irritating potential of surfactants strongly depends on their type [95,96,97,98,99,100,101,102,103]. The lowest Zein Number values were observed for the DG and CB surfactants, which also correlates with their low CMC values. There is a clear correlation between the CMC value and the potential for skin irritation. These results are consistent with the monomer penetration model, which suggests that only surfactant monomers have the ability to penetrate biological membranes. In contrast, micelles, due to their larger size, are either unable to penetrate or do so at a significantly slower rate. Depending on the CMC value, the concentration of free monomers will vary, which affects their rate of penetration. As a result, surfactants characterized by a higher concentration of monomers will penetrate membranes faster than those with a low CMC value [104].

Ionic surfactants exhibit a strong affinity for globular proteins. The charged head group of the ionic surfactant molecule interacts electrostatically with the amino acid groups of proteins that carry an opposite charge. Additionally, the alkyl chain of the surfactant engages in interactions through hydrophobic bonds with non-polar regions on the surface and inside globular proteins. Protein denaturation occurs when the concentration of surfactants exceeds the saturation threshold of the protein [105,106].

The literature has reported a significant correlation between the ability of surfactants to denature proteins and their irritating potential for the skin. This phenomenon can be explained by the interactions of surfactants with proteins, such as keratin, present in the structure of the skin. Therefore, surfactants that do not cause protein denaturation should be classified as non-irritating substances for the skin.

According to the classification and main properties of surfactants, anionic surfactants are characterized by high effectiveness but may exhibit irritating effects. Amphoteric surfactants, such as cocamidopropyl betaine, are generally milder and better tolerated by the skin. Non-ionic surfactants, such as alkyl polyglucosides, exhibit the least irritating potential [107].

All selected surfactants were tested for their effectiveness to extract bioactive compounds from grape pomace. The milled and powdered grapevine pomace were dispersed in micellar extraction medium of different surfactants.

The resulting grape pomace extracts based on the solution of following surfactants: Decyl Glucoside, Cocamidopropyl Betaine, Sodium Coco-Sulfate, and Disodium Cocoyl Glutamate were marked as GPE_DG, GPE_CB, GPE_SCS, and GPE_DSCG, respectively. These extracts were subjected to comprehensive characterization to elucidate the profile and activity of the bioactive compounds isolated from grape pomace through the loan extraction process.

This research focuses on the design and preparation of model natural cosmetics for skin hygiene, considering the empirical verification of their efficacy and safety parameters for the skin. The novel approach represents a remarkable advancement in the field of cosmetics production, emphasizing the potential of loan extraction (LE) and grape pomace in creating natural, effective, and safe cosmetics for skin hygiene.

#### 2.1.2. Microbiological Stability

The obtained extracts were subjected to analysis for their microbiological stability. Microbiological duo plates were used for the tests to count the total number of bacterial colonies, as well as for the cultivation of yeasts, fungi, and molds. As a result of the conducted tests, no colonies of bacteria, fungi, yeasts, or molds were detected. All tested extracts demonstrated the required microbiological stability.

#### 2.1.3. Determination of Selected Compounds by UPLC-MS/MS

In this study, ultra-high-performance liquid chromatography coupled with electrospray ionization–tandem mass spectrometry (UPLC-MS/MS) was employed to qualitatively and quantitatively determine the compounds leached from grape pomace applying the Loan Chemical Extraction process using various extraction media containing different types of surfactants. The results, presented in Table 4, provide a detailed assessment of the compositions of the extracts, with a particular focus on phenolic compounds, amino acids, and sugars.

Among the identified bioactive compounds, catechin and epicatechin were predominant across all extracts. The extraction medium containing the amphoteric surfactant CB facilitated the highest release of these compounds, yielding concentrations of 267 mg/L for catechin and 650 mg/L for epicatechin. Similarly, substantial concentrations of these compounds (218 mg/L catechin and 524 mg/L epicatechin) were observed in extracts obtained using an anionic surfactant DSCG.

These values significantly exceed those reported by Chiavalori et al. [108], as well as findings from other studies utilizing alternative extraction methods. Moreover, they surpass the results obtained by Brazinha et al. [109] in which different combinations of solvent to water with added citric acid were used in the extraction media.

Catechins are known for their numerous health benefits in cosmetic applications, including the scavenging of free radicals generated by ultraviolet (UV) radiation and environmental pollutants. Additionally, they promote collagen synthesis while inhibiting matrix metalloproteinase enzymes [110].

Gallocatechin and rutin were detected in significant concentrations in extracts obtained using the DG-based medium (1.46 mg/L and 0.619 mg/L, respectively), suggesting that this surfactant is particularly effective in facilitating their extraction. Conversely, the concentrations of quercetin (4.78–4.80 mg/L), trans-resveratrol (0.197–0.199 mg/L), and gallic acid (0.191–0.265 mg/L) remained stable across different extraction media, indicating their relative resistance to variations in surfactants. These results are similar with those reported by Sazdanić et al. [111]. A higher concentration of gallic acid was observed by Brazinha et al. [109] when extraction was performed using different solvent-to-water ratios in combination with citric acid. This suggests that the extraction efficiency of gallic acid is influenced by the solvent system composition. The literature extensively documents the anti-inflammatory and antibacterial properties associated with gallic acid [112], further highlighting its potential applications.

Proper skin function requires appropriate care and the use of cosmetic formulations enriched with natural bioactive compounds, including amino acids. Amino acids play a crucial role in skin regeneration and overall condition improvement. One of the most effective methods for delivering these beneficial effects is through external application. Since the skin is continuously exposed to environmental factors, direct application of cosmetic products provides the most effective defense. Cosmetic formulations containing amino acids contribute to enhanced skin hydration and overall skin health [113]. In this study, the highest concentration of amino acids was detected in extracts obtained using a medium containing the non-ionic surfactant DG, with a total concentration of 76.6 mg/L. Decyl Glucoside is widely used in cosmetic formulations due to its mild nature, making it particularly suitable for sensitive skin prone to irritation. Similarly, the surfactant SCS, which exhibited the highest irritancy potential, was associated with a total amino acid concentration of 75.1 mg/L in the GPE_SCS extract. Extracts formulated with GPE_DG may provide a gentle, non-irritating effect on the skin while promoting hydration and regeneration due to their high amino acid content. These findings suggest that the inclusion of such bioactive-rich extracts in cosmetic products may enhance skin barrier function and overall dermal health.

Glucose, being the simplest sugar, was present in high concentrations in all analyzed extracts. The highest concentration of glucose was observed in GPE_SCS (982 mg/L), followed closely by GPE_DG (967 mg/L). The highest fructose concentration was recorded in the GPE_DSCG extract (236 mg/L), while the highest concentration of mannose and very high of D-sorbitol was detected in GPE_DG (respectively, 249 mg/L, 112 mg/L). Notably, the concentration of D-sorbitol remained relatively stable regardless of the extraction medium used. Overall, the extract derived from the surfactant DG exhibited the highest total sugar content.

#### 2.1.4. Total Phenolic Content (TPC) and Antioxidant Capacity (DPPH, ABTS)

One of the goals of this study was to determine total phenolic content and evaluate the antioxidant capacity of grape pomace extracts obtained using aqueous micellar systems as a components for cosmetic formulations. The total phenolic content (TPC) and antioxidant activity, assessed using the DPPH and ABTS assays, were measured for investigated extracts. The results, summarized in Table 5, expressed as mean ± standard deviation (SD), revealed significant variations among the tested extracts. The TPC values demonstrate that the GPE_DSCG medium yielded the highest phenolic content (877.3 ± 25.8 mg GAE/L), followed closely by GPE_DG (856.2 ± 32.2 mg GAE/L). In contrast, GPE_SCS exhibited the lowest TPC (510.1 ± 12.7 mg GAE/L). A similar trend was observed in antioxidant activity. The DPPH radical scavenging activity was highest for GPE_DSCG (1791.1 ± 29.3 mg TE/L), followed by GPE_DG (1480.8 ± 31.3 mg TE/L). Lower activity was observed in GPE_CB (694.5 ± 34.3 mg TE/L) and GPE_SCS (579.8 ± 28.1 mg TE/L). Similarly, in the ABTS assay, GPE_DSCG exhibited the highest antioxidant activity of 2233.7 ± 9.1 mg TE/L, with GPE_DG (of 983.8 ± 10.5 mg TE/L, while GPE_SCS showed the lowest ABTS scavenging capacity (1245.0 ± 15.2 mg TE/L).

These findings emphasize the significant impact of the extraction medium on the efficiency of polyphenol recovery and antioxidant potential. The superior performance of GPE_DSCG and GPE_DG suggests that these media optimize the solubilization and stabilization of phenolic compounds, leading to enhanced extraction efficiency and bioactivity. Hosseinzadeh at al. studied surfactant-water-methanol mixtures at various ratios and found that micellar-water solutions of surfactants exhibited the highest efficiency in polyphenol extraction, which can be attributed to their amphiphilic nature and ability to form various micellar phases in both aqueous and organic solutions [73]. Similarly, Sharma demonstrated that among all the formulations tested for fruit juice extraction, the non-ionic surfactant exhibited the highest extraction efficiency compared to other surfactant formulations and various conventional solvents [114]. Several studies have shown that specific types of non-ionic surfactants have shown better efficiency of polyphenols extraction from various plant-based raw materials than ionic surfactants. Also, Sazdanić et al. highlighted the importance of surfactant selection, showing that non-ionic surfactants generally outperform anionic and cationic surfactants due to their optimal hydrophilic-lipophilic balance (HLB) [115]. Research by Skrypnik et al. [74] and Śliwa [116] further delivers that non-ionic surfactants facilitate polyphenol solubilization and lower surface tension, improving the extraction process. Moreover, Atanacković Krstonošić et al. demonstrated that mixtures of non-ionic surfactants with longer oxyethylene chains exhibit synergistic effects, enhancing the extraction efficiency of less polar polyphenolic compounds [111]. In our study, high results were obtained for both the non-ionic surfactant—DG, and the relatively mild anionic surfactant—DSCG. This makes DSCG an alternative and expands the range of effective extraction agents for cosmetic applications.

### 2.2. Characterization of Cosmetics Products

In the next stage of our research, the impact of the addition of extracts on the basic physicochemical properties of cosmetics was analyzed. For this purpose, a model shower gel (SG_E_10p) enriched with 10% (*w*/*w*) grape pomace extracts was prepared using the concept of Loan Extraction based on borrowed non-ionic surfactant DG. The formulation was designed based on existing literature and our prior experience, aiming to develop a preliminary cosmetic product with potential market interest. The properties of the obtained shower gels (SG_E_10p) were compared with those of a shower gel without added extract (SG_E_0p). The main objective was to verify whether the concept of borrowing a portion of cosmetic ingredients for the extraction process and then returning them, enriched with bioactive compounds extracted from grape pomace, would adversely affect the quality properties of the final cosmetic products. The composition of the compared shower gels is presented in Table 6, and the properties obtained are shown in Table 7.

#### 2.2.1. Sensory Properties

The addition of grape pomace extract to shower gel had a positive effect on its sensory properties. In particular, the extract addition gives it a subtle, natural fragrance reminiscent of fresh grapes and vineyard notes, contributing to a more appealing and pleasant scent profile. Moreover, the presence of polyphenolic compounds in grape pomace has a significant impact on the color properties of the extracts and cosmetic preparations obtained. These compounds determined the color of the final product through their known absorption properties in the visible range. Visual tests have shown that the addition of grape extract to a base shower gel causes a change in its color to light amber. Such color modifications may be important for potential impact on consumers. Users have often reported a gentle, nourishing sensation on the skin, which enhances overall sensory experience.

#### 2.2.2. Stability

**Figure 3:**
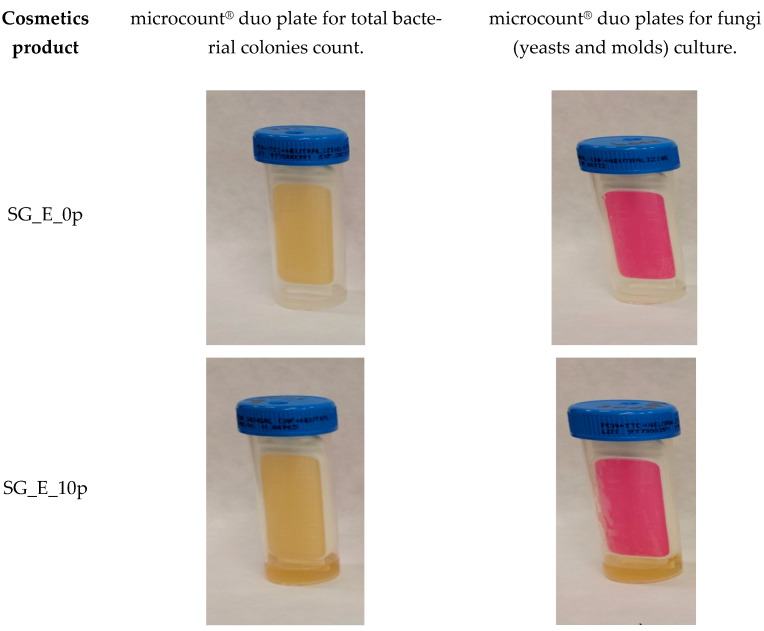
Photos of the duo plates dipslides after 5 days of incubation time.

The mechanical and microbiological stability of the designed cosmetic products was measured. The products remained homogeneous after centrifugation at 5000 rpm (30 min). In addition, a freeze–thaw test was performed by exposing the product to freezing temperatures (−18 °C) for 24 h and then allowing it to thaw at room temperature for 2 h. The sample was then placed at a higher temperature (40 °C) for 24 h and then again at room temperature for 24 h. After three cycles of freeze–thaw testing, no changes in the appearance of the preparations were observed, and the prepared model shower gels were considered stable under these experimental conditions. The shower gels obtained were also analyzed for microbiological stability. For this purpose, double microbiological plates were used to count the total number of bacterial colonies and to culture fungi (mold and yeast). No changes were observed on the tested duo plate dipslides after incubation time of 3 and 5 days (Figure 3). The test results confirmed the required level of microbiological stability for all cosmetic products tested.

#### 2.2.3. Viscosity

In the cosmetics industry, viscosity control is crucial because it has a significant impact on the overall quality of the product, its consistency and optimal dispensing from the packaging. This importance is particularly evident in cosmetic products based on surfactants. The viscosity management process is primarily based on the use of viscosity modifiers, which are used to precisely adjust the final properties of the product in terms of viscosity. The desired viscosity level is achieved not only as a technical requirement but also as a key element that enables optimal dispensing from the packaging and usage. Shower gels are formulated with a relatively high viscosity, typically ranging from 3000 to 6000 mPa·s, to ensure a desirable texture and ease of application. In our research, the same amount of viscosity modifier, sodium chloride, was used to adjust the viscosity to approximately 5000 mPa·s. The results indicated a decrease in viscosity upon addition of the extract (Table 7). The reduction in viscosity is associated with the presence of active ingredients derived from the extract, which affected the gelling ability of the thickening agents in the preparation. However, this decrease does not negatively affect the properties of the designed shower gel.

#### 2.2.4. Foaming Properties

The results of the foaming ability and foam stability tests of the designed shower gels are presented in Table 7. The data obtained indicate that the analyzed preparations were characterized by high values of both foaming ability and foam stability. The introduction of the extract into the formulation resulted in a slight decrease in these parameters; nevertheless, aqueous solutions of the investigated products were able to generate good foam of 500 cm^3^.

#### 2.2.5. Irritating Potential

As demonstrated in Section 2.1.1, depending on the structure and nature of surfactants, they may have an irritating effect on the skin. There are a number of methods for reducing the irritating potential of surfactants in finished cosmetic preparations [1,95,117,118]. Scientific literature indicated that the addition of natural active substances to cosmetics can increase the safety of such products [118]. Empirical studies show that the addition of plant extracts to surfactant solutions can reduce their irritant potential. The presence of polyphenols, flavonoids, proteins and carbohydrates in these extracts may result in integration with the micelles formed by surfactants, causing them to grow and stabilize the structure of the micellar aggregates. Such structural changes contribute to a reduction in the irritant potential of surfactants [1]. In the case of the designed shower gel formulation, a highly beneficial reduction in irritation potential was observed after the addition of 10% (*w*/*w*) grape pomace extracts. However, the formulated shower gel without the extract addition was also classified as non-irritant.

## 3. Materials and Methods

### 3.1. Materials

Analytical standards of (+)-catechin, (+)-Catechin, (−)-Epicatechin, (−)-Gallocatechin, Rutin, Syringic acid, L-Phenyloalanine, L-Aspartic acid, L-Valine, L-Lisyne, L-Tryptophan, L-Leucine, L-Threonine, L-Methionine, L-Histidine, D-(−)-fructose, D-(+)-mannose were purchased from Merck (Darmstadt, Germany), trans-Resveratrol from LGC (Teddington, England), Quercetin and Gallic acid from POL-AURA (Zabrze, Poland), D-(+)-glucose and D-sorbitol from Sulpeco (Pennsylvania, PA, USA).

All standards used were of analytical grade (≥99% purity).

The extraction medium were made using certified, vegetable-based raw materials which are approved for the production of natural products according to ECOCERS and COSMOS standards: decyl glucoside, DG (Plantacare 2000, BASF, Ludwigshafen, Germany), cocamidopropyl betaine, CB (Rokamina K30, PCC-Excol, Brzeg Dolny, Poland), sodium coco-sulfate SCS (Sulfopon 1216 G, BASF, Germany), disodium cocoyl glutamate, DSCG, (Plantapon ACG, BASF, Germany) distilled water.

### 3.2. Plant Material

The pomace from a mixture of Solaris Muscat and Riesling white grapes were obtained from the Cwielong-Olszewski vineyard (Opole Province, Poland). All grapes were harvested at full ripeness (in early September 2024).

### 3.3. Determination of Bioactive Compounds by UPLC-ESI–MS/MS

The grape pomace used for the investigation was obtained from the Cwielong-Olszewski Vineyard, established in 2013 in Balcarzowice, Poland. The grapes were harvested at full ripeness in early September 2024. The grape pomace was destemmed using a destemming crusher. Subsequently, the pomace was pressed in a fruit and wine press. On September 17, 2024, the pomace from white hybrid grape varieties—Solaris, Muscat, and Riesling—was delivered to the laboratory three days after harvesting and pressing.

The pomace was deeply frozen at a temperature of −18 °C until the time of analysis. During the experimental time, the pomace was removed from the freezer and allowed to thaw for 2 h at a temperature of 22 °C, after which it was used directly in the extraction process. The climate in the grape-growing area is temperate, with an average annual temperature of approximately 9.5 °C. The maximum monthly temperature reaches about 19.9 °C in July, while the minimum temperature drops to around −1.5 °C in January. Average annual precipitation is approximately 750 mm, and relative humidity is about 74.4%. For all grape varieties, the berries exhibited a spheroidal shape of medium size, with a diameter of approximately 16 mm. The clusters were loose, with an average cluster weight ranging from 150 to 200 g.

#### Quantitative Analysis of Selected Compounds in Grape Pomace Extracts by UPLC-ESI-MS/MS

Selected compounds were analyzed in independent repeats. The extract solutions was filtered through the 0.2 µm syringe filters and separated using ultra-performance liquid chromatography system, UPLC (Sciex ExionLC AD, AB Sciex, Concord, ON, Canada). A (Kinetex 3.5 µm XB-C18 100 Å; 100 × 4.6 mm, Phenomenex Torrance, CA, USA) reverse-phase pre-column and column maintained at 30 °C was used. A 0.1% (*v*/*v*) aqueous formic acid as solvent A and methanol as solvent B were applied The flow rate of the mobile phase was 0.5 mL/min, the dosing volume was 1 µL. The gradient elution conditions for the analysis with positive-ion mode were set as follows: 0.0–20 min 15–50% B, 20–25 min 50% B, 25.0–25.1 min 50–15% B, 25.1–30 min 15–15% B, while for the method with negative-ion mode: 0.0–10 min 5–5% B, 10–20 min 5–50% B, 20–25 min 50% B, 25.0–25.1 min 50–15% B, 25.1–30 min 15–15% B.

A triple quadrupole mass spectrometer was used for MS detection (4500 QTRAP, AB Sciex Concord, ON, Canada). The ionization source parameters were as follows: ion spray voltage, 4500 V and −4500 V for positive and negative-ion mode, respectively; source temperature, 600 °C; nebulizing gas, 50 psi; drying gas, 50 psi; curtain gas, 35 psi. ANALYST 1.7.2 software was used to automatically optimize the data collection parameters obtained in MRM mode. Accordingly, standard solutions of the individual standards (concentration of 1 ng/mL) were infused directly using an infusion pump. Identification of selected polyphenolic compounds, amino acids and sugars was carried out based on selected MRM pairs and retention times of standard substances, while keeping chromatographic conditions constant. The obtained surface areas of the analyzed substances allowed their quantification, using appropriate calculations.

Instrumental analysis calculations were performed using ANALYST 1.7.2 software with a 1/x weighted linear regression calibration curve, optimized for data points. Calibration curves for all polyphenols were generated from peak areas and standard concentrations, by dilution of standard stock solutions (10 mg in 10 mL methanol) using LC-MS grade methanol. Detector linearity for quantified compounds was demonstrated using calibration standards injected at seven concentrations ranging from 0.1 to 100 μg/mL. The extracts were injected directly without dilution.

### 3.4. Determination of Antioxidant Properties

#### 3.4.1. Total Phenolics Content (TPC)

The total phenolic content (TPC) was measured spectrophotometrically using the Folin–Ciocalteu (FC) method, following Singleton et al. (1999) with slight modifications [119]. A 50 µL of the diluted extract (10-fold dilution with distilled water) was mixed with 200 µL of Folin–Ciocalteu reagent and 600 µL of a 20% sodium carbonate (Na_2_CO_3_) solution, then brought. to 4 mL with distilled water. After 120 min of incubation at room temperature in the dark, the absorbance was measured at 765 nm. TPC was quantified in milligrams of gallic acid equivalent per liter of extract (GAE/L) with triplicate measurements.

#### 3.4.2. Antioxidant Activity (DPPH Test)

The antioxidant activity of the extract was determined using a modified Brand-Williams et al. (1995) method [120]. A 50 µL aliquot of a diluted extract was mixed with 950 µL methanol. Then, 3 mL of a 0.1 mM methanolic DPPH solution was added. After shaking and incubating in the dark at room temperature for 30 min, absorbance was measured at 517 nm against a methanol blank. Results were expressed as mg Trolox equivalents per liter of extract (TE/L), with all tests performed in triplicate.

#### 3.4.3. Antioxidant Activity (Abts Test)

The antioxidant activity of the extract was evaluated using a modified method based on Re et al. [121]. The ABTS●^+^ radicals were prepared by mixing 1 mL of a 0.01 M ABTS (2,2-azino-bis-(3-ethylbenzothiazoline-6-sulfonic acid) diammonium salt] solution with 1 mL of 0.005 M potassium persulfate., then kept in the dark for 20 h. For the assay, 300 µL of each solution (50 µL of extract diluted in 950 µL of distilled water) was mixed with 2 mL of ABTS●^+^ solution. After 6 min, the absorbance was measured at 734 nm using distilled water as a blank. Results were expressed as mg Trolox equivalent per liter of extract (TE/L, with all measurements performed in triplicate.

### 3.5. Preparation of Micellar Extracts from Grape

A 2% (*w*/*w*) aqueous solution of independent surfactants—decyl glucoside (DG), cocamidopropyl betaine (CB), sodium coco-sulfate (SCS), and disodium cocoyl glutamate (DSCG)—was used as the extraction medium. Mixture of Benzyl Alcohol, Benzoic Acid, Dehydroacetic Acid, and Tocopherol in the concentration of 0.5% (*w*/*w*) was used as preservatives. Ground with a laboratory knife mill (Cutter Mixer R5 Plus, Robot Coupe, Vincennes, France) grape pomace (400 g) was mixed with 100 g of the extraction medium and stirred vigorously at 380 rpm for 20 min at room temperature, using a mechanical stirrer (CAT, R50D; M. Ziperer GmbH, Ballrechten-Dottingen, Germany). The resulting extract was filtered under vacuum (Vacuum Pump V-700, Büchi, Flawil, Switzerland) using a Nalgene^®^ bottle-top sterile filter units with pore size 0.45 μm and polyethersulfone membrane (Thermo Fisher Scientific Inc. Waltham, MA, USA), and the filtrate was used for further studies.

### 3.6. Characterization of Cosmetic Product (Shower Gel)

#### 3.6.1. Viscosity

The viscosity measurements of cosmetic products were performed using a Brookfield DV2TRV rheometer (Brookfield, Brookfield, USA) equipped with a small sample adapter and SC4 cylindrical spindle. Each test used an 8 mL sample, and measurements were performed at a temperature of 20 °C and rotational speed of 10 RPM. The measurement was repeated three times.

#### 3.6.2. Foaming Properties

The foaming properties of shower gels were measured in accordance with PN-74 C-04801. The Ross-Miles apparatus measuring cylinder was used to assess the foaming ability of the preparation. A volume of 50 mL of a 10% (*w*/*w*) aqueous solution of the tested shower gels was poured into the cylinder, while 200 mL of the same solution was introduced into the dropper. The height of the foam formed was measured at 1 min and 10 min after the start of the measurement, enabling the evaluation of the foaming ability and foam stability over time.

#### 3.6.3. Determination of Irritant Potential—Zein Value

A 2 g sample of protein was dissolved in 40 g of a 1% surfactant solution or a 10% shower gel solution to prepare the test solutions. The concentration of solubilized protein was determined using Kjeldahl analysis. The results were expressed as milligrams of solubilized protein per 100 mL of the sample. To ensure accuracy and reproducibility, each measurement was performed in triplicate, and the final value was calculated as the arithmetic mean of the three independent measurements. This procedure follows the protocol originally described by Wasilewski et al. [1].

#### 3.6.4. Microbiological Stability

The microbiological stability of the extracts and shower gel was assessed using the Microcount^®^ Duo microbiological testers (Schülke & Mayr GmbH, Norderstedt, Germany). The nutrient media carrier for determining the total plate count uses an agar that enables growth of the most common microorganisms. For sample collection, a sterile swab was used to apply samples onto dipslides. The collected samples were subsequently streaked onto the agar surface of the testing slides to facilitate the growth of microbial colonies.

The microbiological plates were then placed in the designated tester chamber and incubated at a controlled temperature of 28 °C. The incubation period was set to 3 days for bacterial colony and fungal assays, while a longer incubation period of 5 days was allocated for the detection of specifically yeasts and molds. Following the respective incubation periods, the plates were visually inspected for microbial growth.

The enumeration of microorganisms was performed using a standardized template provided by the manufacturer, which ensure accurate quantification of the detected colonies. The detection limit is approx. >100 CFU/mL.

### 3.7. Statistical Analysis

All UPLC-ESI-MS/MS data are presented as the mean of four replicates (n = 4) ± standard deviation (SD). Mean values were analyzed using one-way ANOVA followed by Tukey HDS post hoc test to identify significant differences. Statistical analyses were conducted with Statistica software ver. 10 (StatSoft, Tulsa, OK, USA). A correlation matrix was applied to identify significant correlations among variables. Differences were found to be significant when the p-value was <0.05.

## 4. Conclusions

The cosmetics industry is currently experiencing significant growth, leading to increased demand for new ingredients, especially those of natural origin. Grape pomace, a by-product of wine production, is a valuable source of phytochemicals that can be used in the production of natural cosmetics. This approach not only contributes to the development of the industry, but is also in line with the principles of environmental and economic sustainability. The development of effective extraction methods is the subject of extensive scientific research.

The results of the presented research provide a comprehensive assessment of the potential for applying the concept of loan extraction in the cosmetics industry. Micelle-assisted extraction, using various surfactants, has been investigated as a tool for developing natural cosmetics. The results obtained emphasize the effectiveness and numerous advantages of this method, drawing attention to the bioactive compounds obtained and their antioxidant activity. The designed models of cosmetic products based on the obtained extracts showed a beneficial effect on reducing the irritation potential of the finished products.

The research conducted showed that the application of the loan extraction concept in the production of hygiene cosmetics, using shower gels as an example, enables the production of safe and natural products.

## Figures and Tables

**Table 1:** Target composition of a model cosmetic product (shower gel).

	Ingredient (INCI Name)	SG [%]
1	Sodium Coco-Sulfate	4.5
2	Aqua	to 100
3	Decyl Glucoside	4.5
4	Benzyl Alcohol, Benzoic Acid, Dehydroacetic Acid, Tocopherol	0.5
5	Disodium Cocoyl Glutamate	1
6	Citric Acid	to pH 5.5
7	Parfum	0.3
8	Cocamidopropyl Betaine	2
9	Sodium Chloride	3

**Table 2:** Borrowed components used in the extraction process—composition of extraction medium.

	Ingredient (INCI Name)	GPE_DG	GPE_CB	GPE_SCS	GPE_DSCG
		% (*w*/*w*)			
1	Decyl Glucoside	2			
	Cocamidopropyl Betaine		2		
	Sodium Coco-Sulfate			2	
	Disodium Cocoyl Glutamate				2
2	Benzyl Alcohol, Benzoic Acid, Dehydroacetic Acid, Tocopherol	0.5	0.5	0.5	0.5
3	Aqua	97.5	97.5	97.5	97.5

**Table 3:** CMC, NOI, and Zein Number values of investigated compounds.

Ingredient (INCI Name)	CMC mmol/L	MW g/mol	CMC %, (*w*/*w*)	NOI %	Zein Number mgN/100 mL
Decyl Glucoside	2.2 [88]	320	0.07	100	7.7
Cocamidopropyl Betaine	0.28 [89]	342	0.01	87	33.3
Sodium Coco-Sulfate	8.5 [90]	330	0.28	100	632.1
Disodium Cocoyl Glutamate	10.6 [91]	191	0.20	100	359.1

**Table 4:** Quantification by UPLC-ESI-MS/MS of detected compounds in the studied grape pomace extracts—mean values ± standard deviation (*n* = 4).

	Compound	Quantification/Confirmation Transition	Family	GPE_DG [mg/L]	GPE_CB [mg/L]	GPE_SCS [mg/L]	GPE_DSCG [mg/L]
1	(+)-Catechin	290.9 > 139.0 290.9 > 123.0	flavonols	142 ± 3.88	267 ± 6.56	82.2 ± 7.16	218 ± 4.18
2	(−)-Epicatechin	290.9 > 139.0 290.9 > 123.0	flavonols	283 ± 1.28	651 ± 8.52	164 ± 2.13	525 ± 16.1
3	(−)-Gallocatechin	306.9 > 288.8 306.9 > 163.0	flavonols	1.46 ± 0.06	1.29 ± 0.12	1.19 ± 0.05	1.36 ± 0.15
4	Rutin	608.9 > 299.9 608.9 > 270.9	flavonols	0.619 ± 0.03	0.529 ± 0.002	0.549 ± 0.03	0.588 ± 0.04
5	Quercetin	300.8 > 151.0 300.8 > 179.0	flavonols	4.78 ± 0.001	4.78 ± 0.002	4.80 ± 0.004	4.80 ± 0.002
6	Syringic acid	196.9 > 120.9 196.9 > 181.9	phenolic acid	0.103 ± 0.001	0.116 ± 0.001	0.113 ± 0.001	0.107 ± 0.001
7	Trans-Resweratrol	226.9 > 185.0 226.9 > 143.0	stilbenes	0.197 ± 0.003	0.199 ± 0.001	0.197 ± 0.002	0.199 ± 0.001
8	Gallic acid	168.9 > 124.8 168.9 > 78.9	phenolic acid	n.d. *	0.265 ± 0.004	n.d. *	0.191 ± 0.05
	Sum of phenolic compounds			433	925	253	750
9	L-phenyloalanine	163.9 > 147.0 163.9 > 103.0	amino acid	1.95 ± 0.27	1.89 ± 0.01	2.33 ± 0.03	1.96 ± 0.03
10	L-Aspartic acid	131.8 > 88.0 131.8 > 114.9	amino acid	4.48 ± 0.58	4.53 ± 0.14	4.77 ± 0.42	4.64 ± 0.01
11	L-Valine	118.1 > 72.0 118.1 > 55.0	amino acid	9.80 ± 0.39	8.03 ± 0.11	10.8 ± 0.06	10.2 ± 0.33
12	L-Lisyne	147.1 > 84.0 147.1 > 130.0	amino acid	27.2 ± 0.95	23.7 ± 0.56	28.4 ± 0.06	26.0 ± 0.51
13	L-Tryptophan	205.0 > 188.0 205.0 > 145.9	amino acid	13.4 ± 0.51	15.1 ± 0.12	11.8 ± 0.43	13.7 ± 0.43
14	L-Leucine	132.1 > 86.0 132.1 > 44.0	amino acid	11.4 ± 0.38	8.41 ± 0.05	12.0 ± 0.24	11.3 ± 0.001
15	L-Threonine	120.1 > 74.0 120.1 > 56.0	amino acid	4.54 ± 0.15	4.06 ± 0.001	4.50 ± 0.006	4.24 ± 0.07
16	L-Methionine	150.1 > 103.9 150.1 > 132.9	amino acid	0.268 ± 0.05	0.350 ± 0.004	0.243 ± 0.008	0.370 ± 0.005
17	L-Histidine	156.1 > 110.0 156.1 > 82.9	amino acid	3.50 ± 0.15	1.78 ± 0.07	0.25 ± 0.04	0.51 ± 0.08
	Sum of amino acids			76.6	67.9	75.1	72.9
19	D-(+)-glucose	178.9 > 58.9 178.9 > 88.9	sugars	967 ± 28.3	787 ± 13	982 ± 16	830 ± 12.1
21	D-(-)-fructose	179.9 > 59.0 179.9 > 90.0	sugars	202 ± 14	170 ± 7.01	186 ± 12	236 ± 13
23	D-(+)-mannose	178.9 > 58.9 178.9 > 88.9	sugars	250 ± 26	196 ± 6.50	217 ± 4.50	193 ± 11
24	D-sorbitol	180.9 > 58.9 180.9 > 70.8	sugars	102 ± 0.41	112 ± 5.34	107 ± 0.41	80 ± 5.34
	Sum of sugars			1521	1266	1492	1339

* not detected.

**Table 5:** Total phenolic content and antioxidant activity (DPPH and ABTS) determined in grape pomace extracts.

Grape Pomace Extract	TPC [mg GAE/L]	DPPH [mg TE/L]	ABTS [mg TE/L]
GPE_DG	856.2 ± 32.2	1480.8 ± 31.3	1983.8 ± 10.5
GPE_CB	587. 9± 16.0	694.5 ± 34.3	1676.0 ± 11.8
GPE_SCS	510.1 ± 12.7	579.8 ± 28.1	1245.0 ± 15.2
GPE_DSCG	877.3 ± 25.8	1791.1 ± 29.3	2233.7 ± 9.1

**Table 6:** Final cosmetic formulation (shower gel).

	Ingredient (INCI Name)	[% m/m]	
		SG_E_0p	SG_E_10p
1	Sodium Coco-Sulfate	4.5	4.5
2	Aqua	do 100	do 100
3	Extract	0	10
	*Decyl Glucoside*	*0*	*0.2*
	*Benzyl Alcohol*, *Benzoic Acid*, *Dehydroacetic Acid*, *Tocopherol*	*0*	*0.05*
	*Aqua*	*0*	*9.75*
4	Decyl Glucoside	4.5	4.3
5	Disodium Cocoyl Glutamate	1	1
6	Citric Acid	to pH 5.5	to pH 5.5
7	Benzyl Alcohol, Benzoic Acid, Dehydroacetic Acid, Tocopherol	0.5	0.45
8	Cocamidopropyl Betaine	2	2
9	Sodium Chloride	3	3

**Table 7:** Basic properties of designed cosmetics products.

Cosmetics Product	Stability	Viscosity mPa·s	Foaming Ability cm^3^	Foam Stability %	Irritating Potential mgN/100 mL
SG_E_0p	stable	5184 ± 50	530 ± 25	98	90.7 ± 0.5
SG_E_10p	stable	4224 ± 30	496 ± 20	97	74.2 ± 0.5

## Data Availability

Data is contained within the article.

## Abbreviations

The following abbreviations are used in this manuscript:

SCS	Sodium Coco-Sulfate
DSCG	Disodium Cocoyl Glutamate
DG	Decyl Glucoside
CB	Cocamidopropyl Betaine
GPE	Grape pomace extract
GPE_DG	Grape pomace extract obtained using Decyl Glucoside solution
GPE_CB	Grape pomace extract obtained using Cocamidopropyl Betaine solution
GPE_SCS	Grape pomace extract obtained using Sodium Coco-Sulfate solution
GPE_DSCG	Grape pomace extract obtained using Disodium Cocoyl Glutamate solution
SG_E_0p	Shower gel without extract
SG_E_10p	Shower gel with addition of 10% of extract
